# Spectral analysis and antibacterial potential of bioactive principles of *Sargassum crassifolium* J. Agardh from Red sea of Jazan origin

**DOI:** 10.1016/j.sjbs.2021.06.017

**Published:** 2021-06-11

**Authors:** Mohammed Albratty, Abdullwahab A.M. Bajawi, Thamer M.H. Marei, Md Shamsher Alam, Hassan A. Alhazmi, Asim Najmi, Zia ur Rehman, Sivakumar Sivagurunathan Moni

**Affiliations:** a Department of Pharmaceutical Chemistry, College of Pharmacy, Jazan University, Jazan, Saudi Arabia; b Ethnopharmacology Research Unit, Jazan University, Jazan, Saudi Arabia; c Substance Abuse and Toxicology Research Center, Jazan University, Jazan, Saudi Arabia; d Department of Pharmaceutics, College of Pharmacy, Jazan University, Jazan, Saudi Arabia

**Keywords:** Seaweeds, Brown algae, Bioactive constituents, Antibacterial effect

## Abstract

Seaweeds have been focused as potential and promising resources to develop novel pharmaceuticals. The present study was aimed to investigate the bioactive principles of *Sargassum crassifolium* (*S. crassifolium*) through organic solvents methanol and petroleum ether extractions individually. The present study also extended to determine the antibacterial potentiality of the bioactive principles from methanolic extract (ME) and petroleum ether extract (PEE) of *S. crassifolium* against a set of human pathogenic bacteria. Gas chromatography-mass spectrometry (GC–MS) and Fourier transform infrared spectroscopy (FT-IR) analysis of the ME and PEE were exhibiting unique bioactive constituents. The antibacterial effect of ME and PEE were showed the moderate spectrum of activity when compared to the standard streptomycin disc against the screened human pathogenic bacteria. The bacterial sensitivity to the ME was sequenced as *Bacillus subtilis* > *Pseudomonas aeruginosa* > *Escherichia coli* > *Klebsiella pneumoniae* > *Staphylococcus aureus* > *Streptococcus pyogenes.* Furthermore, the spectrum of activity of PEE was showing more or less similar pattern of action with almost equal potency. The spectrum of activity of PEE extract was in the order Bacillus *subtilis* > *Pseudomonas aeruginosa* > *Escherichia coli* > *Staphylococcus aureus* > *Streptococcus pyogenes > Klebsiella pneumoniae.*

## Graphical abstract

**Figure:**
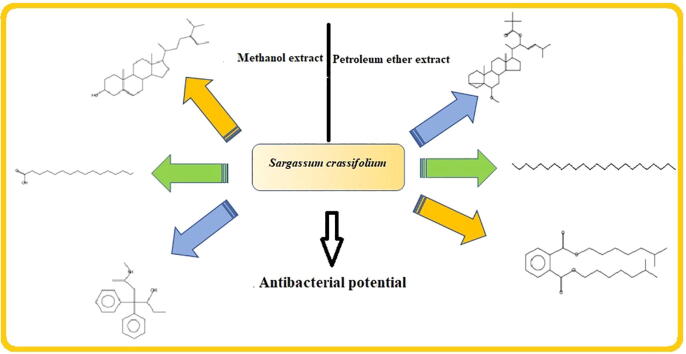

## 1 Introduction

The purpose of finding newer drug molecules, specifically anti-bacterials/anti-microbials, recently getting much attention among various researchers since resistance to antibiotics is a great threat in the ultra-modern therapeutic era (WHO, 2020). Antibiotic resistance is a serious issue globally that necessitates the development of new antibiotics and antibacterial agents. Antibiotic resistance raises the risk of bacterial infections, causing morbidity and mortality (Angel et al., 2021). Natural products are a one-of-a-kind source of bioactive chemicals due to their great molecular diversity and intriguing pharmacological activities. In recent years, drug development has focused to marine natural products, which are a rich source of novel pharmaceutically active molecules (Moni et al., 2018a). Seaweeds are sea algae that live attached to rocks. They are taxonomically varied and may have pharmacological use, particularly as anti-bacterials. (Moni et al., 2018a, Moni et al., 2018b, Maria et al., 2016). The red sea of Jazan, Kingdom of Saudi Arabia enriched with seaweeds especially brown algae (Syeda et al., 2018). In earlier works, we demonstrated the bioactive constituents of *Sargassum aquifolium* (Turner) C. Agardh from the Red Sea, Jazan and their anti-bacterial efficacy (Sivakumar et al., 2019). In continuation of our earlier report (Sivakumar et al., 2019), the present study was designed to determine the active constituents of *Sargassum crassifolium*, a brown alga commonly in the Red sea of Jazan, Kingdom of Saudi Arabia. *S. crassifolium* is considered a good biological indicator of nitrogen sources in urbanized coastal areas (Roldan and Ephrime, 2010). There are minimal research papers published regarding the medicinal value of *S. crassifolium*. The seaweed *S. crassifolium* from the Red sea was not yet explored for pharmaceutical significance. Interestingly, the present demonstrating the anti-bacterial efficacy of bioactive principles extracted through methanolic and petroleum ether extracts from *S. crassifolium* by hot continuous percolation.

## 2 Materials and methods

### 2.1 Study area

**Fig. 1:**
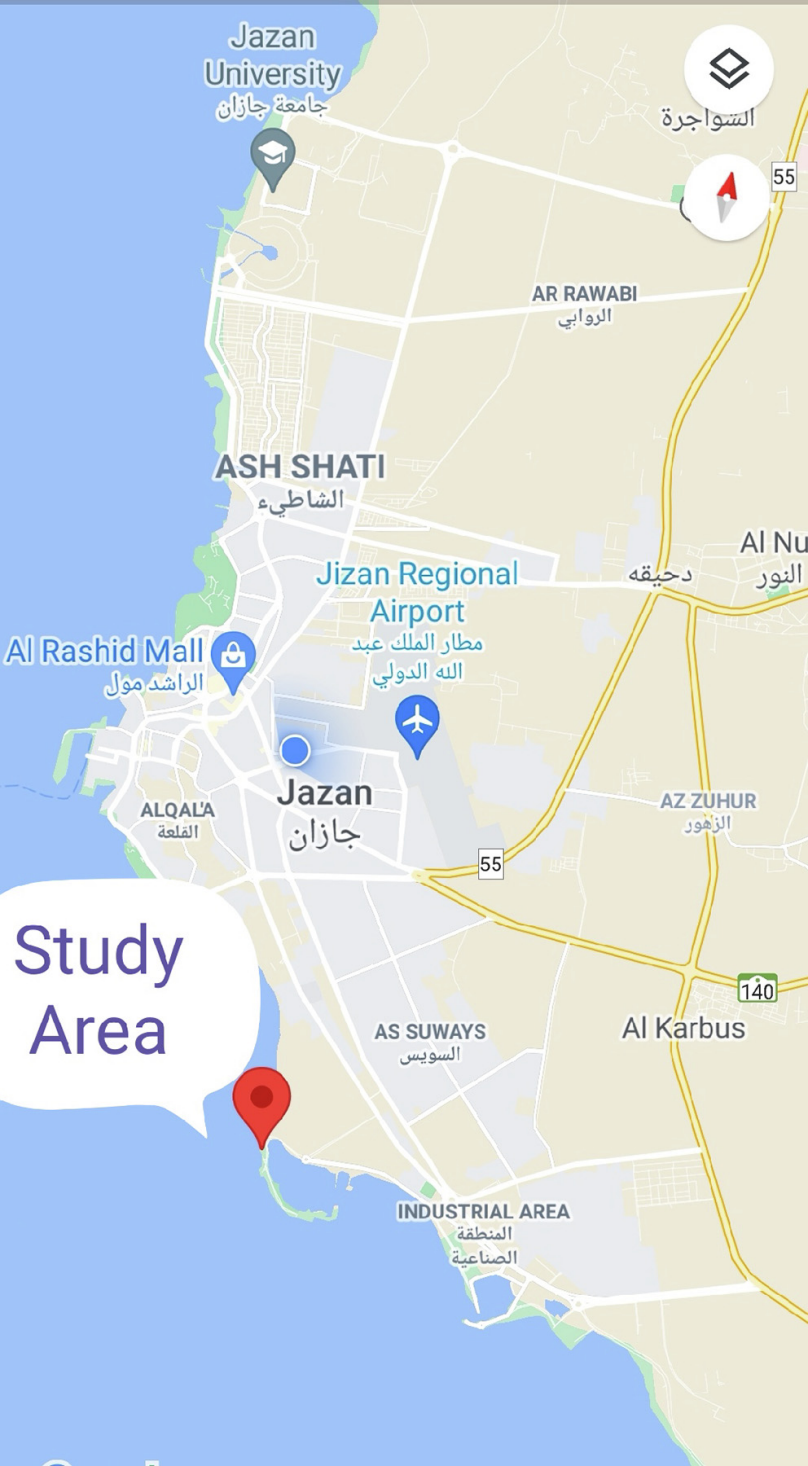
The Study Area Al Murjan beach of Red Sea, located 10 km from the city Jazan, Jizan province, southwest part of Saudi Arabia. The area Coordinates 16° 53′ 21″ North, 42° 33′ 4″ East in direction.

Jazan is the capital city of Jizan province which is located in the southwest part of Saudi Arabia. The city is situated in the coastal region, which is the bed of the Red sea enriched with the natural habitat of varieties of seaweeds, especially brown algae. However, seasonal variations influence the growth of seaweeds (Sivakumar et al., 2019). *S. crassifolium* is present in the Red sea which has not been studied concerning their pharmaceutical significance. The algal specimen was collected from Al Murjan beach which is located 10 km from Jazan city. The map of Al Murjan beach coordinates 16° 53′ 21″ North and 42° 33′ 4″ East (Fig. 1). The seaweed was collected at seashore level by going inside the sea within an area of 10 to 20 m from the seashore.

### 2.2 Processing and identification

**Fig. 2:**
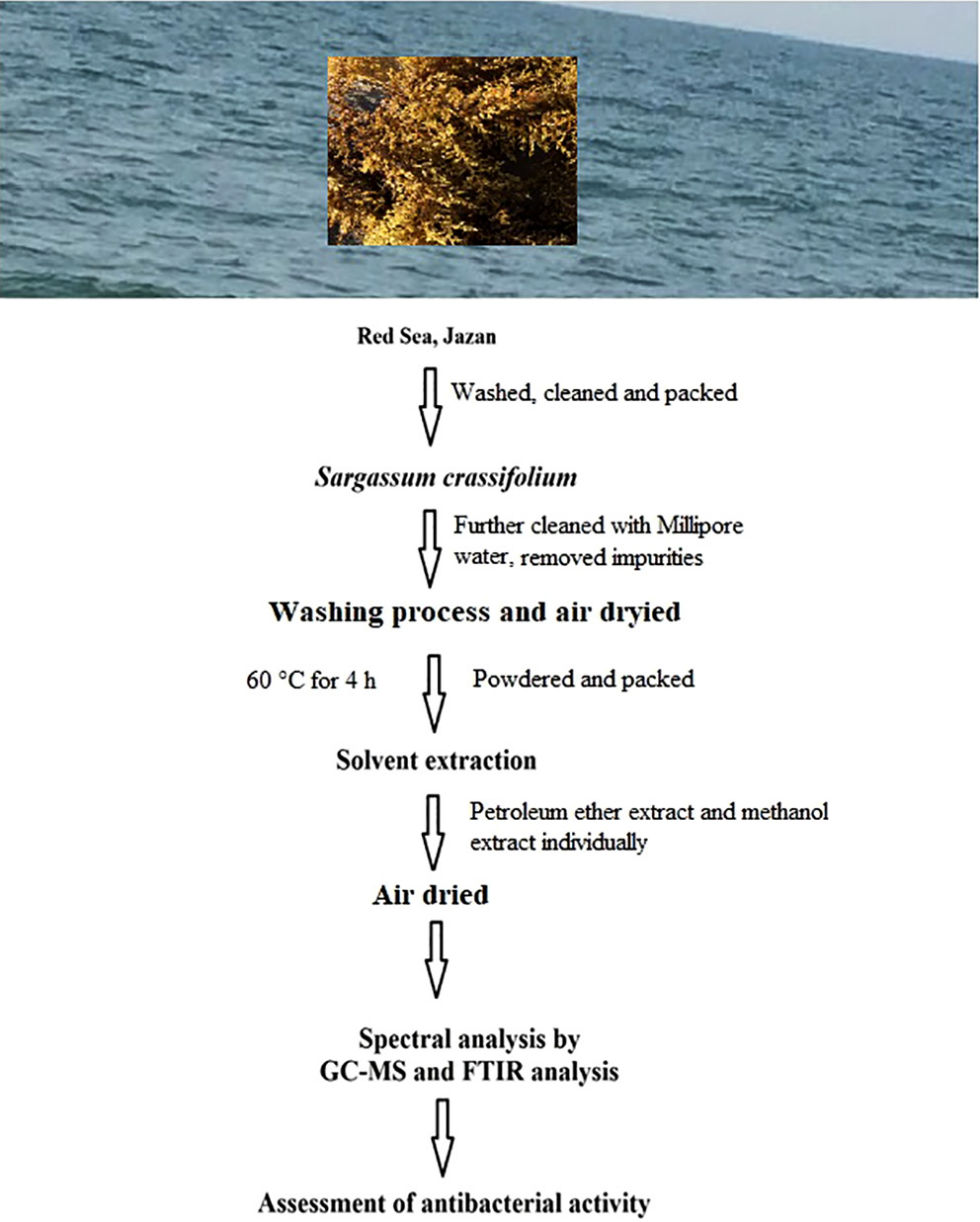
The schematic representation of the experimental work.

The collected seaweed was washed twice thoroughly in seawater at the seashore and the water was drained out by exposing in the open air. Thereafter, the water drained seaweeds were packed in polythene biohazard yellow bags, tied and then transported to the laboratory. Then the samples were thoroughly washed in normal tap water to remove the adhered substances. After washing the seaweed sample was air-dried under the shade and the specimens were identified in the Herbarium of Jazan University (JAZUH), by depositing a voucher specimen. The identification reference number is JAZUH 1630. The complete experimental works scheme is presented in Fig. 2. The washed *S. crassifolium* was air dried by exposing to open air in shade at room temperature for two weeks. The air-dried *S. crassifolium* was finely powdered using a grinder. The powdered samples were pooled and packed in an air-tight container for further experimental uses.

### 2.3 Solvent extraction

Hot continuous percolation was used to extract the active principles from *S. crassifolium* using the Soxhlet apparatus with methanol and petroleum ether individually at 60 ^0^C for 4 h. The extracts were transferred in separate glass beakers for individual solvent extraction and kept open for solvent evaporation through the air-drying process. Following complete air drying of the solvent extracted sample and subjected to GC–MS and FT-IR spectral studies to determine the various compounds present in *S. crassifolium.*

### 2.4 Spectral studies

#### 2.4.1 Gas chromatography-mass spectrometry (GC–MS) analysis

The presence of various bioactive principles of *S. crassifolium* was determined by using Thermo Scientific GC–MS AS 3000 with autosampler and ISQ detector. The structural interpretation of the mass spectrum of bio constituents was identified by using inbuilt software library NIST, Mainlib and Replib.

#### 2.4.2 Fourier transform infrared spectroscopy studies (FT-IR)

The functional groups of the samples were analyzed by using Nicolet iS10 FT-IR spectrophotometer. KBR pellet technique was followed and the spectra of the pellet sample were obtained at 400–4000 cm^−1^ with a resolution of 4 cm^−1^. The presence of specific group and their corresponding compounds in ME and PEE was identified by FT-IR spectroscopy studies and tabulated in Table 1, Table 2.

**Table 1:** FT-IR absorption frequencies (cm^−1^), intensity estimation and functional group of methanol extract of *Sargassum crassifolium.*

Wave number (cm^−1^)	Intensity Estimation	Group or Functional Class	Nature of Functional group	Type of vibration	Possible Compounds
3370	W	N-H, O-H	Amines, Alcohols	Stretching	Phenols, Amino acids Polysaccharides
2946	S	C-H	Alkane (CH_3_ and CH_2_)	Asymmetrical Stretching	Aliphatic compounds, Steroids, Tannins, Saponins
2833	W	C-H	Alkane (CH_3_ and CH_2_)	Symmetrical stretching	Aliphatic compounds
2524	W	S-H	Thiol	Stretching	Amino acids
2219	W	C-O, P-H	Phosphine	Stretching	Phosphine
1655	S	C = O,C = C, N = O	Ester, Nitrate	Asymmetrical Stretching	Carboxylic acid,Ester, Pectin
1449	S	C-H	Methyl	Bending	Tannins / Cutin/Steroids
1023	S	S = O	Sulfonides	Stretching	Starch and Polysaccharides

S – Strong; M- Medium; W- Weak

**Table 2:** FT-IR absorption frequencies (cm^−1^), intensity estimation and functional group of petroleum ether extract of *Sargassum crassifolium.*

Wave number (cm^−1^)	Intensity Estimation	Group or Functional Class	Nature of Functional group	Type of vibration	Possible Compounds
2958	S	C-H	Alkane	Stretching	Aliphatic compoundsSteroids, Tannins,Saponins, Steroids
2926	M	C-HO	Aldehyde/ketones	Stretching	Aliphatic compounds
2873	W	C-H	Alkane (CH_3_ and CH_2_)	Symmetrical stretching	Aliphatic compounds
2360	W	C-O, P-H	Phosphine	Stretching	Phosphine
1464	M	C-H, O-H	Phenolic alcohol	Bending	Tannins / Cutin/Steroids
1378	M	S = O	Sulfone	Stretching	Alkanes
1272	W	C-O	Phenols	Stretching	Tannins / Cutin
1107	S	C-N	Aliphatic amine	Stretching	Aliphatic compound
888	S	C-H	1,2,4tri substituted	Bending	Glucose/Galactose,Tannins
818	W	N-H	Amines	Out of plane Wagging	Fatty acids
738	M	C-Cl	Alkyl halides	Stretching	Halogenated compounds

S – Strong; M- Medium; W- Weak

### 2.5 Antibacterial studies

A set of human pathogenic bacteria of both Gram-positive and Gram-negative bacteria was screened in this study as established by Sivakumar and Safhi, 2013. The bacterial strains used in the study were *Staphylococcus aureus, Streptococcus pyogenes, Bacillus subtilis, Klebsiella pneumoniae, Escherichia coli and Pseudomonas aeruginosa*. Briefly, 24 h culture was prepared and standardized by gradient dilution from 10^-1^ to 10^-7^ with nutrient broth. The screened bacterial culture viability was identified by determining colony forming unit in 1 mL (CFU/mL). The antibacterial susceptibility test was performed by employing Muller Hinton (MH) agar plates. The agar well diffusion technique was used to evaluate the antibacterial potential of both ME and PEE of *S. crassifolium*. The concentration of both ME and PEE were fixed predetermined at 200 µg/ mL for the determination of antibacterial activity. 100 µL of standardized culture was placed on agar plates individually and the culture was spread uniformly on agar plates. The plates were kept for 30 min to allow the bacteria culture to diffuse through the media. Wells of 10 mm in diameter was made by punching on the agar plates using sterilized standard borer. The extracts were placed in the respective wells and the plates were incubated at 37 °C for 24 h. The antibacterial efficacy was accessed by determining the zone of inhibition and the values were expressed in millimeter (mm). Kirby Bauer technique was performed to determine the antibacterial potentiality of standard streptomycin disc (10 µg/disc) (John et al., 2018, Sivakumar et al., 2019). The plates were incubated at 37^°^ C for 24 h. The antibacterial spectrum was assessed by the development of inhibitory zones around the discs after 24 h of incubation. The spectrum of activity is directly proportional to the diameter of the zones of inhibition and tabulated in Table 3.

**Table 3:** Antibacterial potential of the bioactive components extracted from *Sargassum crassifolium.*

Bacterial Organisms	Concentration of 24 h culture CFU /mL	Methanolic extract	Petroleum ether extract	Streptomycin10 mcg/disc
*Bacillus subtilis*	3 × 10^-5^	18.83 ± 1.5	19.5 ± 1.4	26.24 ± 1.6
*Staphylococcus aureus*	3 × 10^-5^	9.5 ± 1.5	10.33 ± 1.9	23.26 ± 2.1
*Streptococcus pyogenes*	4 × 10^-4^	7.6 ± 1.2	8.3 ± 0.6	22.6 ± 1.3
*Escherichia coli*	2 × 10^-6^	15.8 ± 0.8	12.2 ± 0.3	28.2 ± 1.6
*Pseudomonas aeruginosa*	3 × 10^-3^	15.16 ± 1.3	14.3 ± 1.6	25.3 ± 1.3
*Klebsiella pneumoniae*	2 × 10^-3^	11.3 ± 1	7.6 ± 1.2	27 ± 1.4

# Each value is the mean of 6 batches with standard deviation, Both the test values are significantly lesser effect when compared to standard streptomycin disc.

## 3 Results

**Fig. 3:**
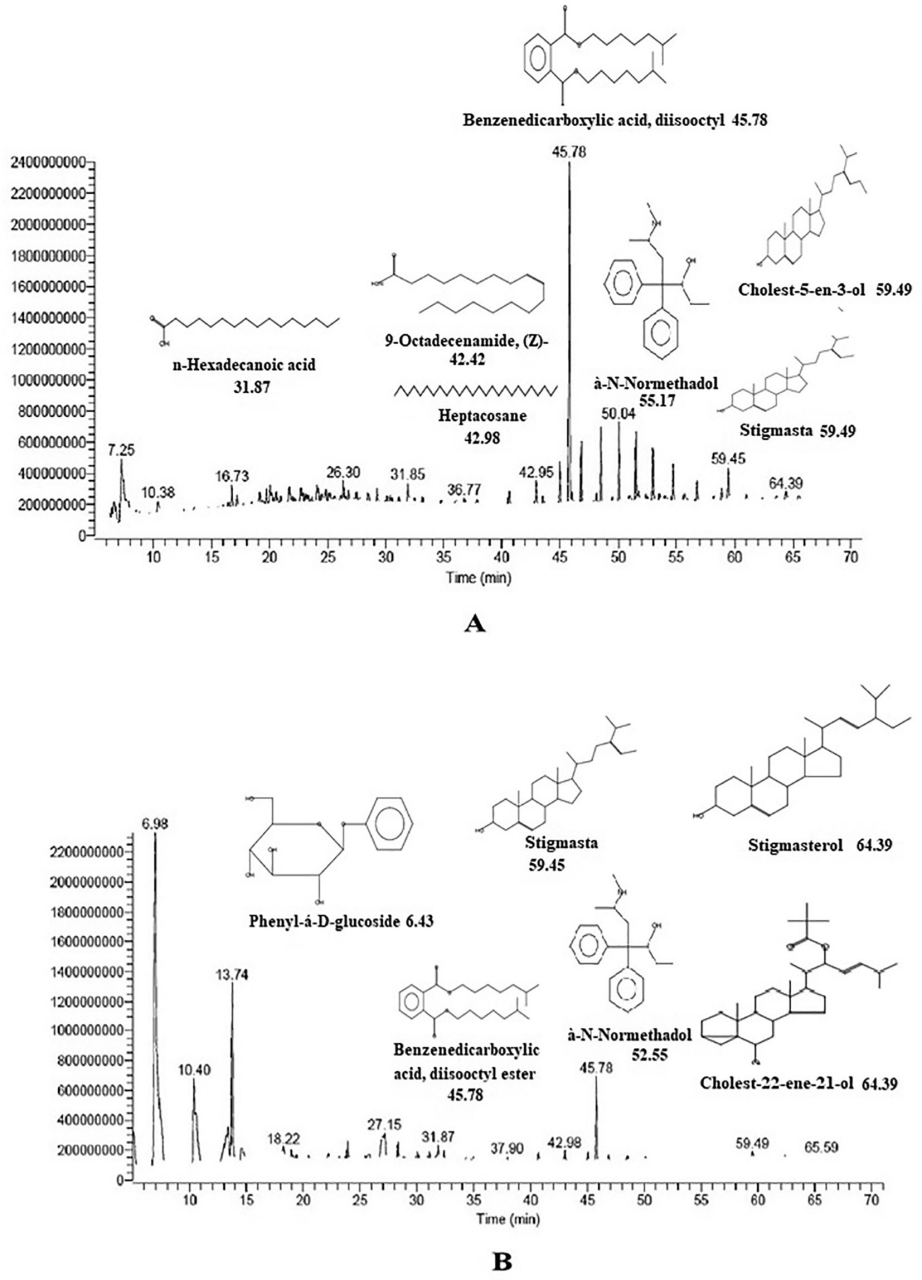
The GCMS chromatogram study of organic solvent extraction *Sargassum crassifolium* (A) Chromatogram of methanolic extract (B) Chromatogram of Petroleum ether extract.

**Fig. 4:**
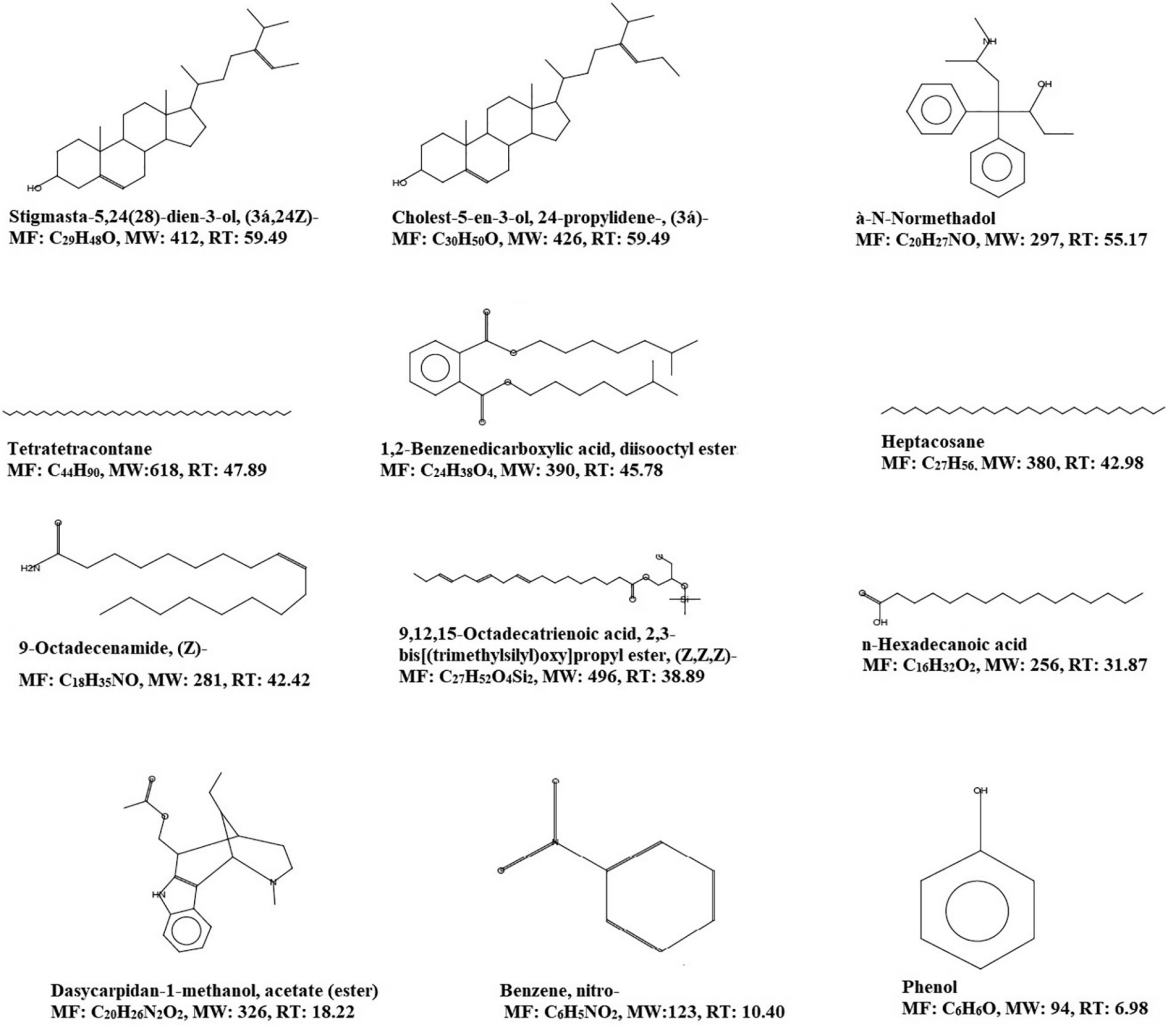
Bioactive compounds of methanolic extract of *Sargassum crassifolium* by GC–MS spectral analysis, *MF: Molecular formula; MW: Molecular weight; RT: Retention time in minutes.

**Fig. 5:**
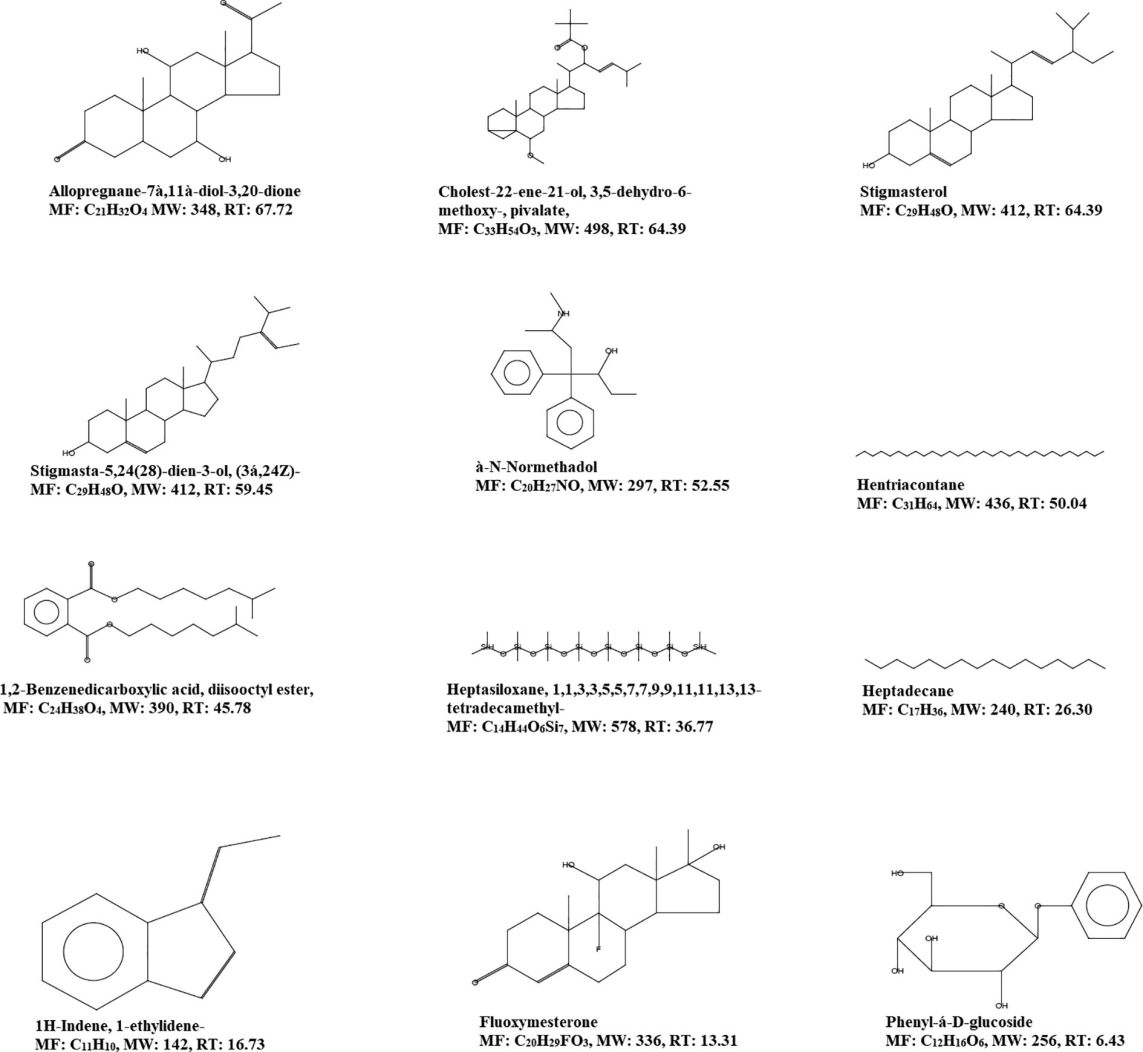
Bioactive compounds of petroleum ether extract of *Sargassum crassifolium* by GC–MS spectral analysis, *MF: Molecular formula; MW: Molecular weight; RT: Retention time in minutes.

**Fig. 6:**
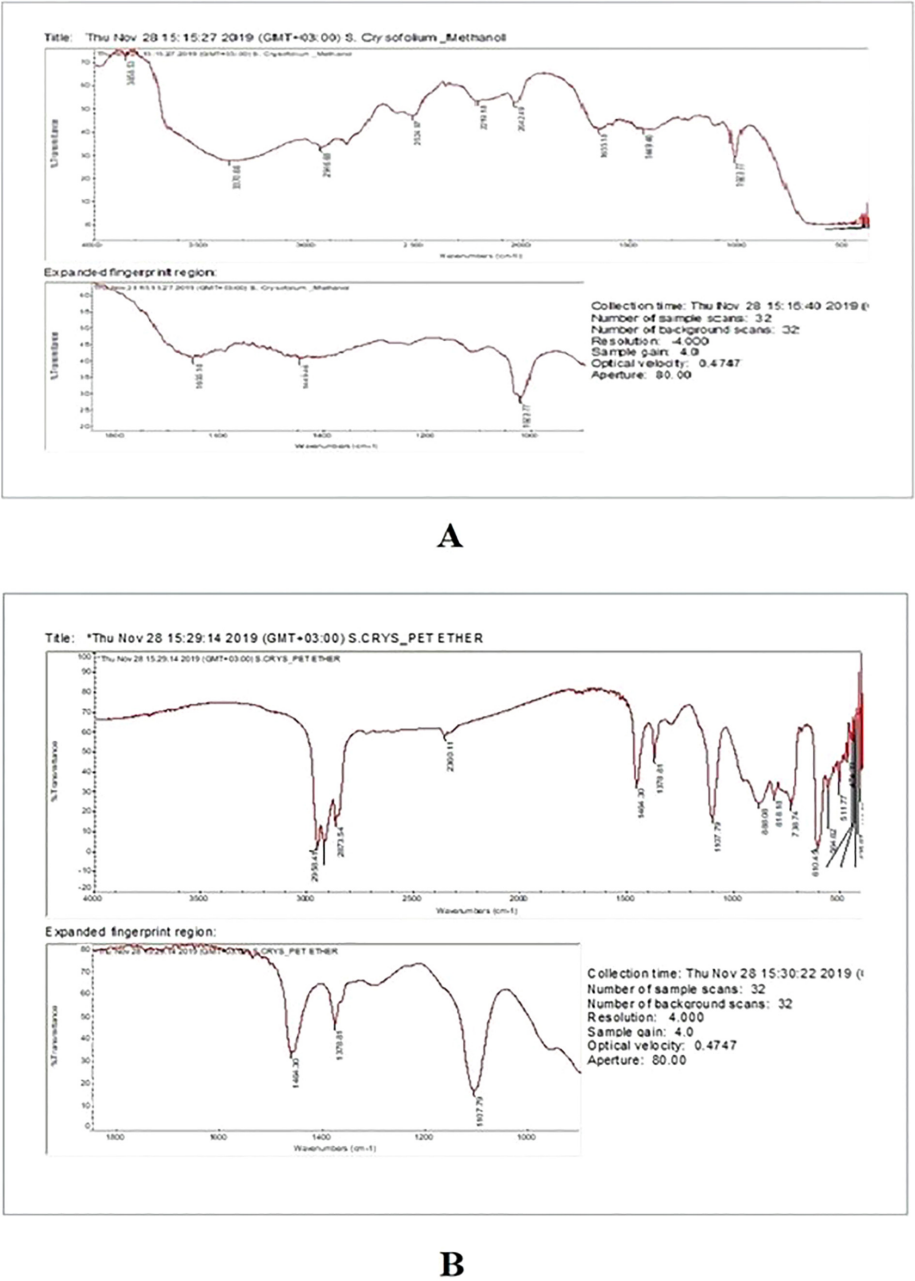
FT-IR spectroscopy study of the organic solvent extracts at 400–4000 cm^−1^ (A) FT-IR spectroscopy of methanolic extract of *Sargassum crassifolium* (B) FT-IR extract of petroleum ether extract of *Sargassum crassifolium*.

The study area Al Murjan beach, Jazan was picturized (Fig. 1) and validated by coordinating 16° 53′ 21″ North, 42° 33′ 4″ East in direction. The flow chart representing the scheme of total experimental work (Fig. 2). The seaweed *S. crassifolium J. Agardh* was collected at the seashore level and processed as described earlier. The GC–MS chromatogram of ME and PEE of *S. crassifolium* was showing various phytoconstituents (Fig. 3 **A & B**). The detected bioactive compounds of both ME and PEE were represented in Fig. 4, Fig. 5 with their possible structure which was instrumental generated by referring in built libraries. Fig. 6.

Interestingly few compounds were commonly present in both ME and PEE namely Stigmasta-5,24(28)-dien-3-ol, (3á,24Z)-, 1,2-Benzenedicarboxylic acid, diisooctyl ester, and à-N-Normethadol. However, the retention time was different form each extract. A unique peak at 6.98 min showing the presence of phenol with 49.61 probability index (Fig. 3**A**). The ME showed Stigmasta-5,24(28)-dien-3-ol, (3á,24Z)- and Cholest-5-en-3-ol, 24-propylidene-, (3á)- exhibited the maximum retention time of 59.49 min followed by 1,2-Benzenedicarboxylic acid, diisooctyl ester was detected at 45.78 min. Heptacosane is an alkane hydrocarbon identified at 42.98 min, however the probability index was 15.82.

Tetratetracontane is a long-chain alkane that was found in the methanolic extract at a retention period of 47.89 min. n-Hexadecanoic acid is otherwise called palmitic acid, a saturated fatty acid was detected at 31.87 min with a probability index of 56.40. In PEE (Fig. 5) was exhibiting various steroidal derivatives such as Allopregnane-7à,11à-diol-3,20-dione, a steroidal derivative was showing the highest retention time of 67.72 min. Followed by Stigmasterol, a steroidal derivative characterized by the presence of hydroxyl group in position C-3 of the steroid molecule. Cholest-22-ene-21-ol, 3,5-dehydro-6-methoxy-, pivalate also a steroidal derivative eluted at a high retention time of 64.39 min. Stigmasta-5,24(28)-dien-3-ol, (3á,24Z)- a phytosterol was detected at the retention of 59.45 min. However, some of the steroidal molecules were also detected at low retention time having more probability index (Fig. 3 **A**). Fluoxymesterone is an anabolic steroid molecule that has been detected at 13.31 min retention time but the probability index was 53.74 as per inbuilt library replib. Heptadecane is an alkane hydrocarbon and hentriacontane is a long-chain alkane hydrocarbon that was observed at 26.30 min and 50.04 min retention time respectively. Phenyl-á-D-glucoside is a glycoside molecule that was detected at 6.43 min retention time but showed 24.48 probability index.

The FT-IR spectra of ME and PEE of *S. crassifolium* were showing unique components (**Fig. 6 & 7**). The ME of *S crassifolium* using the hot continuous percolation method showed various phytoconstituents such as amino acids, saponins, starch, polysaccharides, tannins, steroids and, cutin in methanolic extract shown in Table 1. However, PEE showed the predominant phytoconstituents as steroids, tannins, saponins, glucose, galactose, fatty acids and halogenated compounds (Table 2).

The peak IR band of the extract observed at 3370 cm^−1^ stretching corresponds to phenolic O-H and N-H groups. Other observed strong intensity peaks represent at 2958, 2946 cm^−1^ (C-Hstr.), 1655 cm^−1^ (C = O str, N = Ostr.), 1107 cm^−1^ (C-Nstr.), 1023 cm^−1^ (S = Ostr), 888 cm^−1^ (CHbend) and 610 cm^−1^ (C-Sstr). Some medium intensity peaks are found at 2926 cm^−1^ (C-H str. aldehyde/ketones), 1464 cm^−1^ (C-H, O-H bend), and 1378 cm^−1^ (S = O str). Peak at 1272 cm^−1^ (phenolic C-Ostr.), 2360, 2219 cm^−1^ (P-Hstr) is of weak intensity peaks.

Table 3 summarizes the antibacterial action of ME and PEE of *S. crassifolium* against selected human pathogenic microorganisms. PEE was found to be more effective against Gram-positive bacteria than ME. In contrast ME was exhibiting a better spectrum of activity than PEE against Gram-negative bacteria. Despite having a broad spectrum of activity against the bacterial pathogens tested, the extract's efficacy was significantly lesser than the standard streptomycin disc (10 µg/disc).

## 4 Discussion

Seaweed has the potential to be used as a resource to isolate and create novel medicinal compounds (Sivakumar and Safhi, 2013, Safhi et al., 2015). *Sargassum crassifolium* J. Agardh a brown alga was collected near seashore in the red sea, Jazan, Saudi Arabia. The GC–MS analysis of ME and PEE revealed the presence of various bioactive compounds. The ME of *S. crassifolium* exhibited the unique compound Stigmasta-5,24(28)-dien-3-ol, (3á,24Z)-, that showed the maximum retention time. Similar kinds of the report have been reported in the petroleum ether extract of *Sargassum aquifolium* (Sivakumar et al., 2019). Cholest-5-en-3-ol, 24-propylidene have been identified in the ME of *S. crassifolium*. Erwan Plouguerne ´et al., 2006 isolated Cholest-5-en-3-ol, 24-propylidene from the red alga *Grateloupia turuturu*. n-Hexadecanoic acid is otherwise called palmitic acid, a saturated fatty acid has been identified as significant biomolecule in ME of *S. crassifolium*. An earlier report suggested that palmitic acid has potential antibacterial and anti-oxidant properties (Ehsan Ehsan and H.Z.E., Ali, G., Mahdi, E. , 2015, Karimi et al., 2015). In 2017 a research report study suggesting that hexadeconic acid was the major fatty acid in *Sargassum granuliferum* which prevents the biofilm forming bacteria (Kamriah et al., 2017). Dasycarpidan-1-methanol, acetate is an alkaloid was observed in the ME of *S. crassifolium*. The compound exhibiting anti-inflammatory, anti-bacterial, anti-fungal and anti-cancer (Abeer Fauzi et al., 2017). Phenol was observed in the ME of *S. crassifolium* which has been reported for its anti-bacterial properties (Celis et al. 2011). In their study demonstrated that phenolic compounds like phenol from the extracts of *Anacardium excelsum* exhibited antibacterial effect. The FT-IR study demonstrated the presence of special peaks of prominent functional groups supporting bioactive mixtures particularly tannins and steroids. In 2014 Kannan reported by on FT-IR analysis of *Sargassum wightii* from Gulf of Mannar, India.

Steroidal derivatives were the major compounds in PEE of *S. crassifolium*. Earlier reports suggesting that stigmasterol is an unsaturated phytosterol have shown various pharmacological activities such as anti-osteoarthritic (Gabay et al., 2010) and anti-tumor (Ghosh et al., 2011). Amina Yusuf in 2018 reported the antibacterial potential of stigmasterol isolated from the stem bark of *Neocarya macrophylla*. The present study showed that the PEE was showing good activity against Gram-positive bacteria. An earlier study suggested that the ethanolic extract of *S. crassifolium* has shown the presence of alkaloid, glycoside, polyphenol, saponin, and volatile oil and demonstrated prominent activity against *S. aureus*. Phenyl-á-D-glucoside, a glycoside has been observed in PEE of *S. crassifolium*. It has been demonstrated as antibacterial properties (Md. Ekramul Islam et al., 2002). 1,2-Benzenedicarboxylic acid, diisooctyl ester was determined in the PEE exhibited good retention time. Studies suggested that the 1,2-Benzenedicarboxylic acid, diisooctyl ester isolated from the unripe fruits of *Nauclea latifolia* reported as an anti-bacterial effect against Gram-positive bacteria *Staphylococcus aureus* and *Bacillus subtilis*. An earlier study reported that the ethanolic extract of *S. crassifolium* exhibited an antibacterial effect against Gram-positive bacteria *Staphylococcus aureus* but showed resistant against Gram-negative bacteria Escherichia coli, fungal strains such as fungi *Candida albicans* and *Aspergillus niger (*Armin and Maribel, 2014). Interestingly, the present study demonstrated that the ME and PEE of *S. crassifolium* were found to have a moderate spectrum of action against Gram-positive and Gram-negative bacteria.

## 5 Conclusion

The present study has documented the scientific evidence of the antibacterial properties of the methanolic (ME) and petroleum ether (PEE) of *S. crassifolium,* a brown alga of Red sea, Jazan Province, Kingdom of Saudi Arabia. Bioactive molecules were widely variant with its molecular structure in both ME and PE extracts. The ME of *S. crassifolium* showed specific compounds with high retention time namely, Stigmasta-5,24(28)-dien-3-ol, (3á,24Z)-, Cholest-5-en-3-ol, 24-propylidene, à-NNormethadol, Tetratetracontane, 1,2-Benzenedicarboxylic acid, diisooctyl ester, Heptacosane, 9-Octadecenamide, (Z)-, and n-Hexadecanoic acid. The PEE of *S. crassifolium* exhibited specific compounds with high retention time namely, Cholest-22-ene-21-ol, 3,5-dehydro-6-methoxy-, Allopregnane-7à,11à-diol-3,20-dione, Stigmasterol, Stigmasta-5,24(28)-dien-3-ol, (3á,24Z)-, à-N-Normethadol, and Hentriacontane. Both the extracts were showed a good spectrum of antibacterial activity. The results of this study were demonstrating the presence of various bioactive compounds that might be beneficial in the development of new drugs especially as an antibacterial agent in the future.

## Declaration of Competing Interest

The authors declare that they have no known competing financial interests or personal relationships that could have appeared to influence the work reported in this paper.
